# Questionnaire and LGBM Model for Assessing Health Literacy levels of Mongolians in China

**DOI:** 10.1186/s12889-022-14392-2

**Published:** 2022-11-05

**Authors:** Yan Hong, Xiaoda Zhang

**Affiliations:** 1 School of Nursing, Inner Mongolia Minzu University, 028000 Tongliao, China; 2 Micron Intelligent Manufacturing Systems Science and Technology (Beijing) Co., Ltd, 100086 Beijing, China

**Keywords:** Health literacy, Assessment model, LGBM regression model, Questionnaire design, Quantitative analysis

## Abstract

**Background:**

It is difficult to accurately assess the health literacy(HL) level of Mongolians by using Chinese conventional HL questionnaire, due to their particularity in language, culture and living environment. Therefore, it is very important to design an exclusive HL questionnaire for them. In addition, the existing statistical models cannot meet the requirement of HL assessment with high precision, so it is necessary to study a new HL assessment model.

**Methods:**

A HL questionnaire with 68 questions is designed by combing the HLS-EU-Q47and the characteristics of Mongolians in China. 742 Mongolians aged 18 to 87 in Inner Mongolia of China answered the questionnaire. A data set with 742 samples is constructed, where each sample has 68 features and 1 target. Based on it, the XGB and LGBM regression models are respectively constructed to assess the HL levels of respondents, and their evaluation effects are compared. The impact of each question on the HL level is quantitatively analyzed by using the feature-importance function in LGBM model to verify the effectiveness of the questionnaire and to find the key factors for affecting HL.

**Results:**

The HL questionnaire has the high reliability, which is reflected by the high internal consistency (Cronbach’s coefficient=0.807) and test-retest reliability (Mutual Information Score= 0.803). The validity of the HL questionnaire is obtained by solving KMO and Bartlett Spherical Test Chi-square Value, which are 0.765 and 2486 ($p<0.001$), respectively. $R^2$ index and the absolute error obtained by using the HL assessment model based on LGBM are 0.98347 and 11, which are better than ones by applying the model based-XGB, respectively. The quantitative analysis results show that all 68 questions have influence on HL level, but their degree are different. The first three factors are age, salary level, the judgment ability for the HL information in media, respectively. The HL level distribution of the respondents was 66.71$\%$ excellent, 25.74$\%$ good and 7.54$\%$ poor, respectively.

**Conclusions:**

The presented HL questionnaire with 68 questions and LGBM regression model can obtain the HL level assessment results with high precision for Mongolians in China. The impact of each question in the questionnaire on the final assessment results can be quantified by using the feature-importance function in LGBM model, which is better than the existing qualitative analysis methods.

## Background

Health literacy (HL) was a complex and multidimensional concept related to literacy [1]. It was defined as “an ability that people maintain and promote themselves health by acquiring, understanding, and using health information” by International Union for Health Promotion and Education [2]. Higher-level HL also included critical thinking, analysis, decision-making, and problem-solving in health-related matters. It was important to improve HL, because it not only could promote peoples’ heathy and application awareness in health services but also could reduce the risk of disease, and thus reduce social burden. It was necessary that public libraries with empowerment, equitable, inclusive, collaborative and integrated characteristics were constructed to improve the HL of individuals, communities, organizations and countries [3].

In recent years, the HL had been widely investigated, in which interviewees included adults, adolescents, children, and patients. The low HL and medication literacy were main risk factors for health of children and adults [4]. A cross-sectional study in [5] showed that about 28-38 ones among 120 students had poor health literacy. It was concluded in [6] that adolescents with psychological symptoms and low HL had non-suicidal risks, which would be solved by intervening their mental health and behavior problems. The digital HL survey for college students found that the students with different educational levels had the different usage levels for digital HL [7].

The relationships between HL and some diseases were investigated widely. It was discovered that the high digital HL could increase the number of cancer survivors [8]. A systematic review for HL in individuals at risk for alzheimers dementia was developed in [9], and concluded that alzheimer’s disease patients couldn’t use HL skills, so it was very important to analyze alzheimer’s disease patients’ needs, and to give them some essential information, because it could help them to make decisions during specific medical situations. By investigating the echinococcosis-specific HL of the Tibet Plateau in China, it was found that the echinococcosis-specific HL was a key factor to prevent echinococcosis [10]. The importance of HL for preventing or curing borderline personality disorder [11], hypertension [12], chronic disease [13], atherosclerosis, ischemic heart disease [14], and type II diabetes mellitus [15] were also investigated, respectively. However, the above studies used the statistics methods.

Information technology was used as a data collection tool applied to HL [16–19], but it wasn’t found that information technology was applied to assess HL level. However, with the rapid development of information science, big data analysis and machine learning technology had been applied widely in medical field to solve statistical problems. For example, the early detection of breast cancer based on CNN and light gradient boosting machine (LGBM) [20], the recognition of cancer cells in blood based on GBDT algorithm[21], classifing and predicting for the survival probability of patients with cancer comorbidities by LGBM [22], analyzing for 12 characteristics of breast cancer by random forest methods [23], and diagnosis for thyroid cancer, colon cancer, liver cancer by SVM approach [24], and predicting disease progression of breast cancer by XGBoost (XGB) [25], and so on. Therefore, we think that it is a good idea that big data analysis and machine learning technique were used to predict or assess HL for an individual or group and quantitatively analyze the effectiveness of every factor on their hearth literacy level.

China has 56 ethnic groups, in which the Mongolian is an Ethnic minority, and it accounts for 8.89% of the total population in China. It is difficult to accurately assess their HL level by using existing Chinese conventional HL questionnaire, due to the particularity of language, writing and living environment of ethnic minorities, Therefore, it is very important to design an exclusive HL questionnaire and assessment model for them.

Therefore, 742 Mongolian in Inner Mongolia, China are surveyed in this paper. The HL questionnaire with 68 questions, the LGBM assessment model with high precision, and a quantitative analysis method for every question are presented.

The innovations of this paper are as follows:

i) From four dimensions, the HL questionnaire with 68 questions is designed by both improving the HLS-EU-Q47 and analyzing Mongolian’s characteristics in Inner Mongolia, China. Four dimensions include health concepts and knowledge literacy, healthy lifestyle and behavior, and healthy skills, as well as health status and disease history.
ii) The data set based on the HL questionnaire is constructed, and the LGBM HL assessment model is presented, which can obtain the higher assessment accuracy than the presented XGB HL assessment model and the statistical models.
iii) The impact of each question in the questionnaire on the HL level is quantitatively analyzed one by one by using the feature-importance function in LGBM model in order to verify the effectiveness of the questionnaire and to find the key factors for affecting HL levels.
iv) The above approaches can provide a new idea for investing HL level of other ethnic minorities in China or ethnic minorities in other countries.

## Methods

### Design and setting

The cross-sectional study was carried out for a period of six months between November 2018 to April 2019 in Inner Mongolia, China. The participants was Mongolian, over 18 years old, with no history of psychiatric disorders, and able to read and comprehend the Chinese language. Each participant introduced with the informed consent, upon their approval, the health literacy questionnaire was provided. 742 participants were invited to the survey. The baseline characteristics of participants are shown in Table 1.

**Table 1:** Baseline characteristics of participants

Characteristics	Participants ($\%$)	Men ($\%$)	Women($\%$)
Age group			
18-30	67(9.0)	29(8.1)	38(9.9)
31-40	286(38.6)	108(30.1)	178(46.5)
41-50	164(22.1)	94(26.2)	70(18.3)
51-60	164(22.1)	93(25.9)	71(18.5)
$\ge$61	61(8.2)	35(9.7)	26(6.8)
Total	742 (100)	359(100)	383(100)
Education(Edu)			
Higher Edu.	88(11.9)	49(13.6)	39(10.2)
Vocational Edu.	447(60.1)	201(56.0)	246(64.2)
Secondary Edu.	105(14.2)	58(16.2)	47(12.3)
Elementary Edu.	80(10.8)	41(11.4)	39(10.2)
Not schooled	22(3.0)	10(2.8)	12(3.1)
Marriage			
Married	606(81.7)	302(84.1)	304)79.4)
Not married	85(11.5)	33(9.2)	52(13.6)
Divorced	51(6.8)	24(6.7)	27(7.0)
Occupation			
State sector	392(52.9)	171(47.6)	221(57.7)
Private sector	322(43.4)	179(49.9)	143(37.3)
Retired	28(3.7)	9(2.5)	19(5.0)
Health insurance			
National	651(87.7)	318(88.6)	333(86.9)
Private	91(12.3)	41(11.4)	50(13.1)
Living with			
Alone	93(12.5)	41(11.4)	52(13.6)
Family	614(81.8)	302(84.1)	312(81.4)
Relatives	35(4.7)	10(4.5)	19(5.0)

### HL questionnaire design

The health literacy assessment survey questionnaire is a tool to assess the health literacy level of respondents, which is designed by a health organization or researchers. The survey questionnaire have a lots of questions, and each question is assigned a score. The health literacy levels of respondents are decided by the respondents’ scores. It is very important to design a suitable survey questionnaire for the health literacy assessment. The HLS-EU-Q47 was developed in 2011, which has 86 questions and mainly investigated the peoples’ abilities to understand the health-related issues and to get the health-related knowledge in the complicated situations caused by the inadequate health literacy. However, the HLS-EU-Q47 can’t be applied directly to Chinese. It is necessary to design a health literacy assessment survey questionnaire. In order to reduce participants’ workload, we combined some similar items in the HLS-EU-Q47, such that 86 items are changed as 47 items. And according to the characteristics of Monggolians in Inner Mongolia, China, 21 questions are added. Therefore, we construct a HL survey questionnaire with 68 questions from four dimensions. Four dimensions include health concepts and knowledge literacy, healthy lifestyle and behavior, and healthy skills, as well as health status and disease history. 68 questions are divided into three parts. The first part is the questions about the respondents’ general situation, which are age area, gender, and territory; The second part is the respondents’ own health-related questions, namely health status and disease history; The third part is the health-related issues. Some similar items among 86 items in the EHLSQ are combined as 47 items in order to reduce the workload of participants. 40 questions in the 47 questions are put in the third part, and others are given in the second part. The scoring method for each question are:

i) Age, height, and weight of respondents are recorded according to their actual values.
ii) The scores in the other 65 items of respondents are assessed by using a five-point, self-reported Likert type scale, such as very easy, fairly easy, fairly difficult, very difficult, and unknown . The lowest score is 1, and the highest score is 5.

The score for each of the 68 questions is added to produce the HL level score for individual.

### Reliability and validity of the HL questionnaire analysis

According to the HL questionnaires completed by 742 Mongolians, the reliability of the designed HL questionnaire is analyzed by calculating the Cronbach’s $\alpha$ value and the Mutual Information Score. They can be obtained by using the Python programs designed by ourselves.

The validity of the HL questionnaire is verified by solving the KMO value and Bartlett Spherical Test Chi-square of the HL questionnaires completed by 742 Mongolians. The KMO value and Bartlett Spherical Test Chi-square can be obtained by using a Python program designed by ourselves.

### Data set construction

According to the HL questionnaires completed by 742 Mongolians, the following data set is constructed.

1 $$\begin{aligned} \Xi&=\{(\varpi _{i}^1, \varpi _{i}^2, \cdot \cdot \cdot , \varpi _{i}^{68}, H_{i})\} \nonumber \\&for\;{} i=1,2,\cdot \cdot \cdot , 742 \end{aligned}$$

where $\Xi$ describes the data set that have 742 samples with 68 features. where *i* is the the $i^{th}$ sample, $\varpi _{i}^1$-$\varpi _{i}^{68}$ are 68 characteristics of the $i^{th}$ sample; $H_{i}$ is the target value of the $i^{th}$ sample, and $H_{i}$ describes the scores obtained by the $i^{th}$ respondent.

### XGB and LGBM model construction

**Fig. 1:**
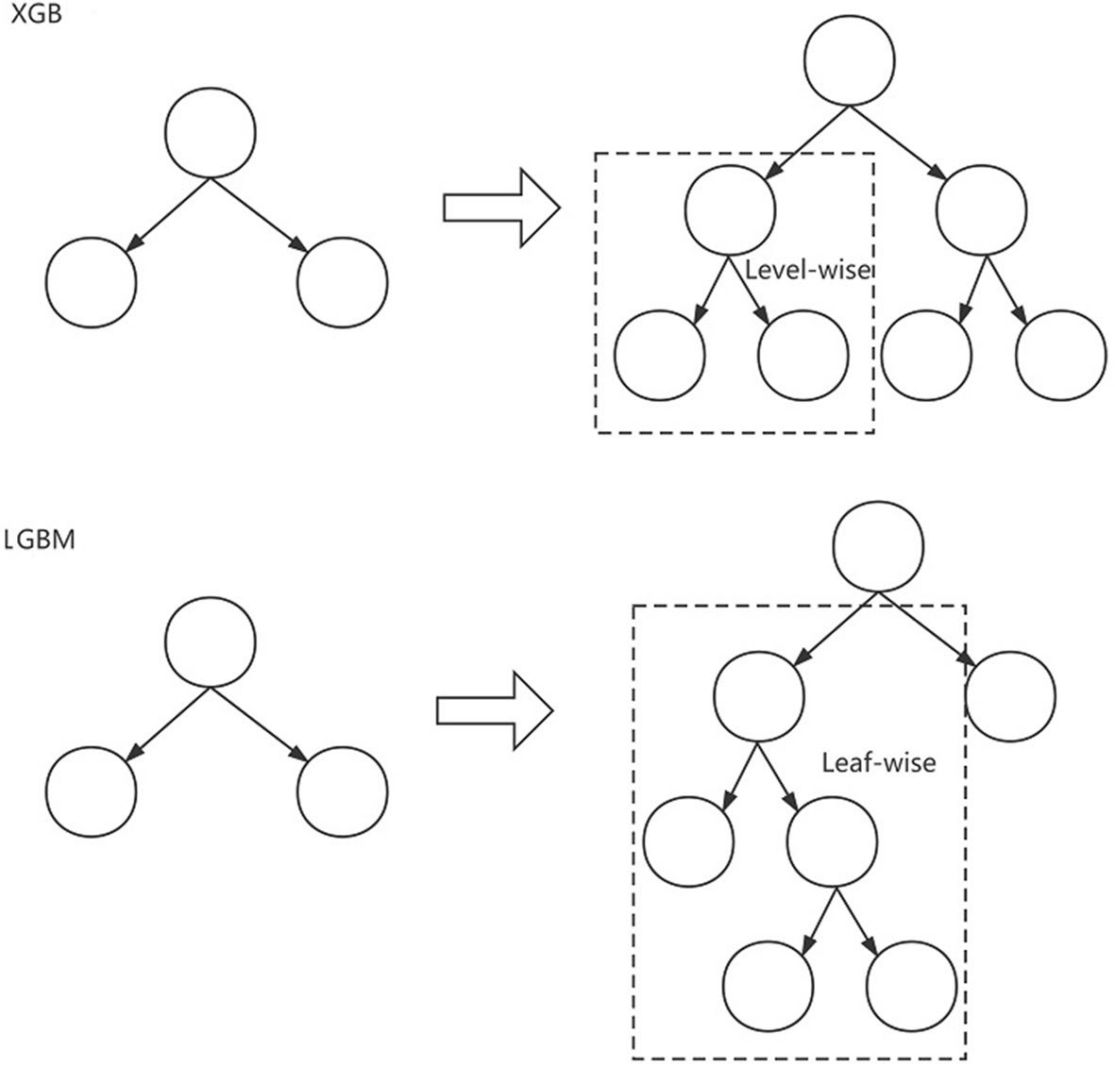
The difference between level-wise and leaf-wise

The GBDT, XGB and LGBM are machine learning models. The XGB model developed from the GBDT model. Compared with the GBDT model, the XGB uses the second-order Taylor expansion for the loss function, such that the prediction accuracy is improved. However, there is lower efficiency in the features selection and growth of the decision tree due to XGB uses hierarchical leaf node selection method. In order to solve the problem, the Histogram algorithm and growing leafs with maximum split gain method were applied in LGBM model, which can improve greatly the prediction accuracy and efficiency. In addition, the maximum depth limit is added to the growth of the algorithm, which can avoid over-fitting under guaranteeing the high training efficiency. Therefore, XGB and LGBM regression models are constructed to assess the HL levels of respondents in this paper. The comparison diagram of XGB and LGBM is shown in the Fig. 1.

The following evaluation index is considered when the LGBM model is applied to predict the considered target, which is called as $R^2$ index.

2 $$\begin{aligned} R^2=1-{\frac{\sum (y_{pred}-y_{true})^2}{\sum (y_{true}-\overline{y})^2}} \end{aligned}$$

where $y_{pred}$ is the predicted value, $y_{true}$ is the true value, and $\overline{y}$ is the average value of the samples. $0<R^2<1$, and a large $R^2$ value indicates a high prediction accuracy.

**Fig. 2:**
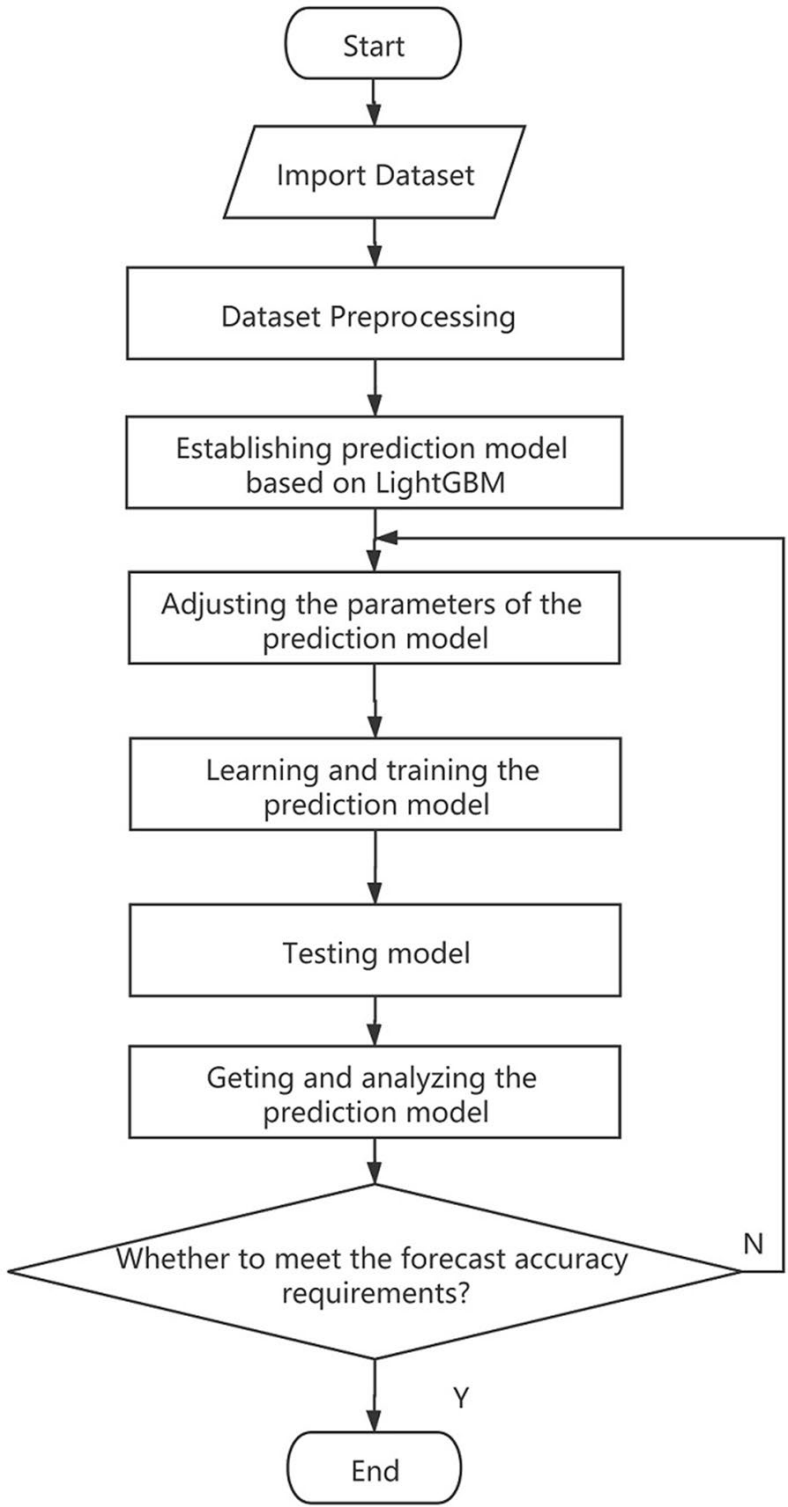
Flow chart of health literacy prediction model

Based on the data set 1, the XGB regression model and LGBM regression model to assess the HL for 742 respondent are constructed, respectively. 80% samples in the data set $\Xi$ are designed as training samples, and others are looked as testing samples. The flow diagram to evaluate HL by using LGBM regression model is given in Fig. 2.

### Quantifing the impact of each question on the final assessment results

By using the ‘feature-importance’ function in LGBM model, we analyze quantitatively the influences of 68 features on the HL assessment results, and find the key factors to affect HL level of Mongolian in Inner Mongolia, China.

The above all processes are completed by our own Python programming.

## Results

### Reliability and validity of the HL questionnaire results

According to the HL questionnaires completed by 742 Mongolians, we calculate the Cronbach’s $\alpha$ value and the Mutual Information Score to analyze the reliability of the designed HL questionnaire. We have obtained that the Cronbach’s $\alpha$ is 0.807 and the Mutual Information Score equals 0.803 by using the Python programs designed by ourselves. It can be seen that the designed HL questionnaire has the high reliability.

In order to verify the validity of the HL questionnaire, the KMO and Bartlett Spherical Test Chi-square Value of the HL questionnaires completed by 742 Mongolians are solved by using a Python program designed by ourselves., which are 0.765 and 2486 ($p<0.001$), respectively. These results show that the designed HL questionnaire has good validity.

### HL assessment results

The HL assessment models based on XGB and LGBM are trained and tested by training samples and testing samples, respectively. $R^2$ indexes obtained by XGB and LGBM regression models are shown in the following Table 2, respectively. From Table 2, it can be seen that the LGBM regression model has more higher HL assessment accuracy than the XGB regression model, and its $R^2$ value is 0.98347, which can meet the actual demands for the HL evaluation.

**Table 2:** $R^2$ indexes obtained by the health literacy prediction models based on XGB and LGBM

Model	XGB	LGBM
$R^2$ Score	0.97553	0.98347

**Fig. 3:**
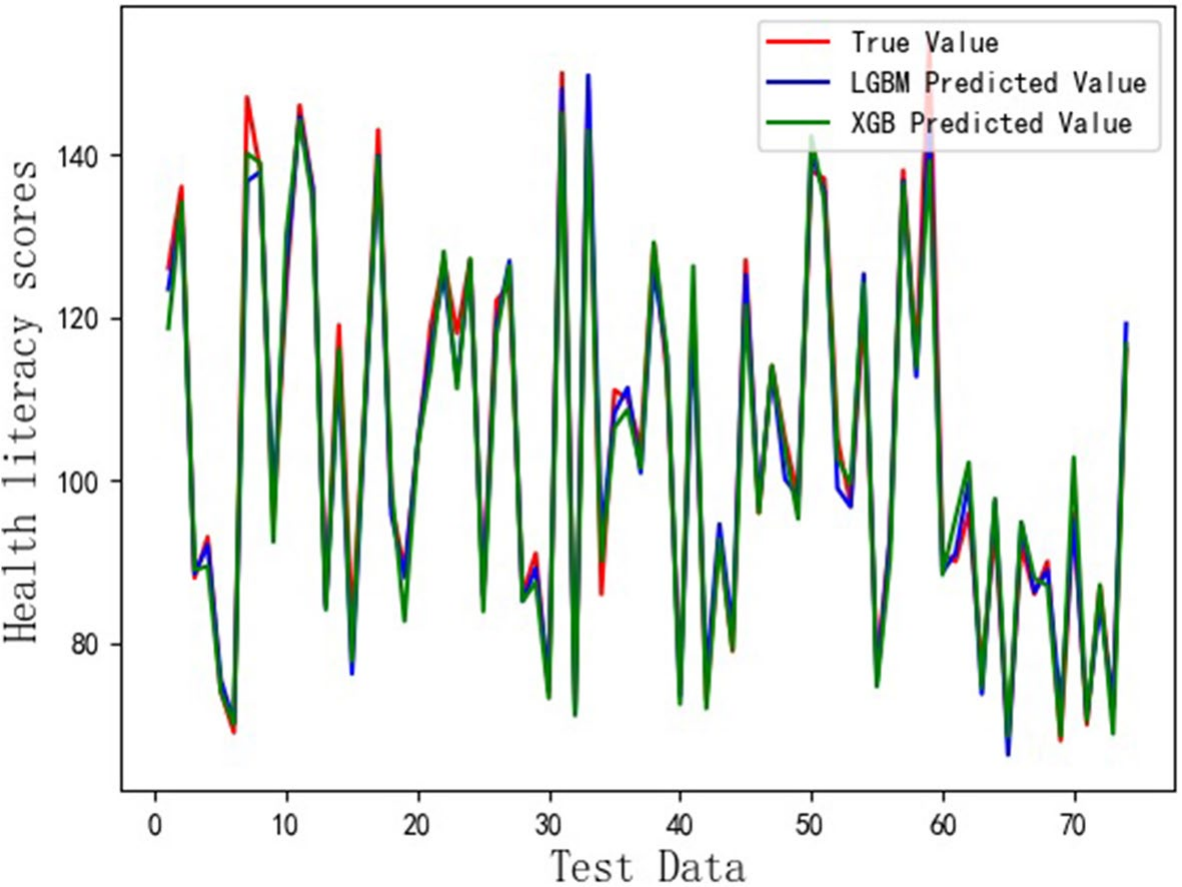
HL scores assessed by using XGB and LGBM

**Fig. 4:**
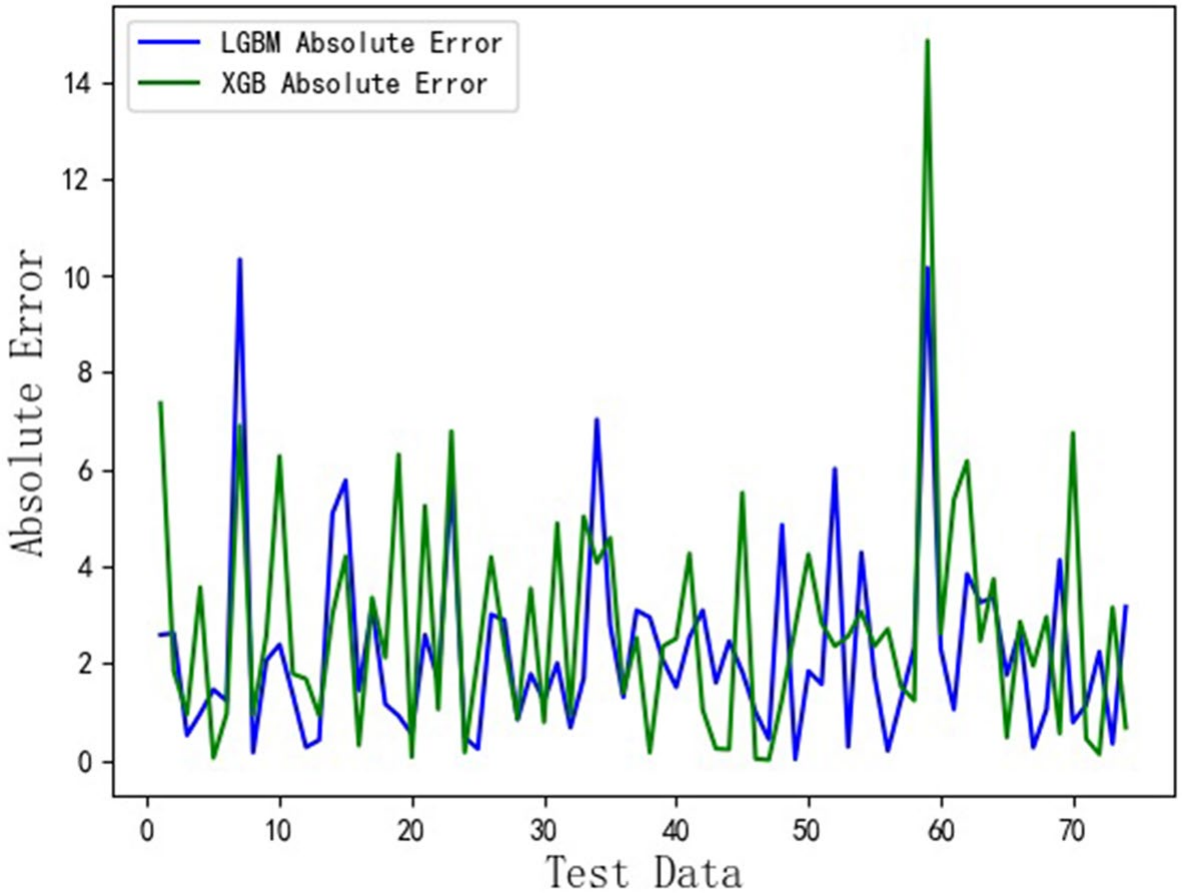
Absolute error rate between the HL assessment results and true values

The Fig. 3 shows that the comparison between the results predicted by XGB and LGBM regression models and the true values. The red line describes the true values; The blue line represents the values predicted by LGBM; The green line is the results predicted by XGB. From the Fig. 3, we know that the high prediction results can be obtained by using XGB and LGBM, respectively. However, the prediction errors from two models can’t be found. Therefore, we draw the absolute error curves obtained by using XGB and LGBM, which are given in Fig. 4. In Fig. 4, the blue line represents the absolute errors between the values predicted by LGBM and the true values. The green line is the absolute errors between the values predicted by XGB and the true values. It can be seen from Fig. 4 that the absolute errors between the values predicted by LGBM and the true values are less than 11, while the absolute errors between the values predicted by XGB and the true values are less than 15. Therefore, the health literacy prediction model based on the LGBM is more effective than one based on the XGB.

**Fig. 5:**
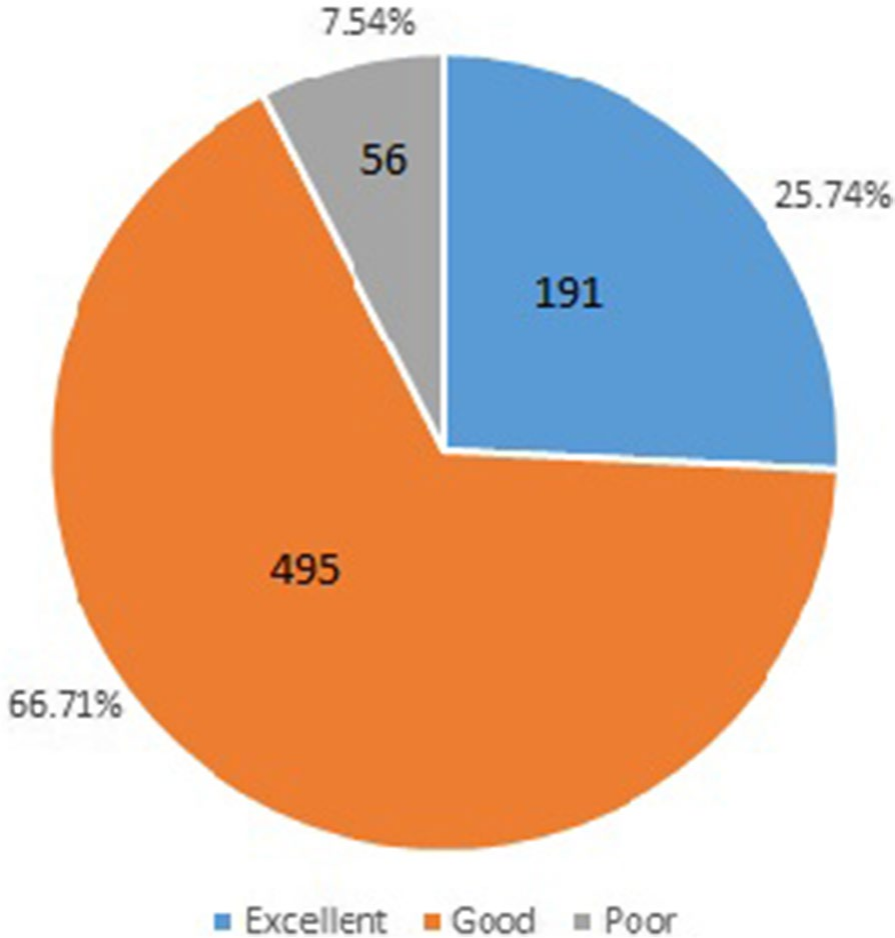
HL level distribution of the respondents

The HL level distribution of the respondents was 66.71$\%$ excellent, 25.74$\%$ good and 7.54$\%$ poor, respectively, which are given in Fig. 5. The percentage of men respondents who scored good and excellent is 58.2 $\%$ , which is better than that of women respondents( 28.1 $\%$ ). The HL scores of the urban respondents are higher than ones of the rural residents. In addition, we find that there is a positive linear correlation between the level of HL and the educational background of the respondents.

### Calculating and analyzing the influences of each question on HL assessment result

**Fig. 6:**
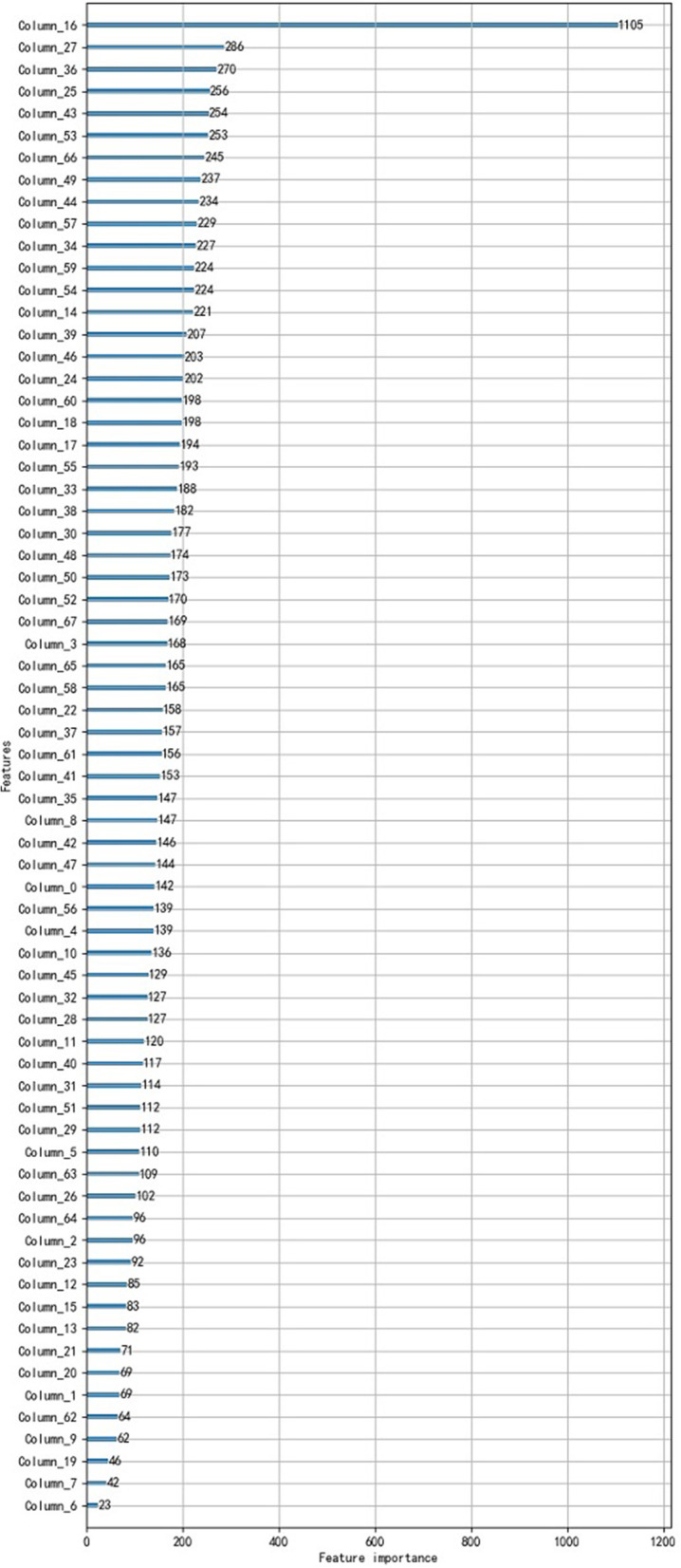
The influence of 68 features on health literacy

The influence of each question in 68 questions on the HL assessment results is respectively calculated by the ‘feature-importance’ function in LGBM, which are shown in Fig. 6. It can be seen that the biggest impact factor is 1105, and the smallest impact factor is 23. The numbers in Fig. 6 are dimensionless. The $Column_{-}16$ (age of the respondents) has the highest influence on the HL level. The $Column_{-}27$ (the salary level of the respondents) is second. The $Column_{-}36$ is third, which is the ability of the interviewees to judge relevant health information in the media. The forth factor, the fifth factor, and the sixth factor are $Column_{-}25$ ( probability of medical attendance), $Column_{-}43$ (knowing about vaccinations and checkups), and $Column_{-}53$ (obtaining healthy eating information), respectively. The influence of Gender ($Column_{-}1$) on the HL level is 69. The impact indexes of the Territory ($Column_{-}2$), Education background ($Column_{-}20$), and Professional ($Column_{-}21$) are 96, 69, and 71, respectively. The forth dimension (health status and disease history) of the HL questionnaire is reflected by the $Column_{-}3,4,5,7,8,9,10$ in Fig. 6, where the impact index of the health status ($Column_{-}3$) is the largest, which is 168. The least influence on the final health index is the $Column_{-}6$, which describes the insurance type used by the respondents. According to the above analysis, it also can be seen that all questions in the designed questionnaire are reasonable, because that they affect the HL assessment results by varying degrees.

The above results can be summarized as:

i) The reliability and validity of the designed HL questionnaire are high, which are respectively verified by ‘Cronbach’s $\alpha = 0.807$’, ‘Mutual Information Score=0.803’, ‘KMO =0.765’, and ‘Bartlett Spherical Test Chi-square Value = 2486 ($p<0.001$)’ .
ii) According to the HL questionnaires completed by 742 Mongolians, the data set with 742 samples and 68 features is constructed to provide. data basis for the HL assessment model based on LGBM or XGB.
iii) Both LGBM-based HL assessment model and XGB-based HL assessment model can accurately predict the HL levels of respondents, and the former’s accuracy is higher than that of the latter, which is 0.98347. Therefore, LGBM-based HL assessment model can be used as an intelligent tool to predict people’s HL levels, which can decrease greatly manual calculations.
iv) Assessment results obtained by applying LGBM-based HL assessment model show that the HL levels of the Mongolian in Inner Mongolia, China are high. Because 92.45% of the respondents have scored above the level of Good, according to Fig,5. The percentage of men respondents who scored good and excellent is 58.2 $\%$ , which is better than that of women respondents( 28.1 $\%$ ). The HL scores of the urban respondents are higher than ones of the rural residents. There is a positive linear correlation between the level of HL and the educational background of the respondents.
v) The influences of each question in the HL questionnaire on the HL assessment results are quantitatively calculated by the ‘feature-importance’ function in LGBM. The results reveal the following points:
  It can be seen that the impact index of ‘Age’ is biggest, and the impact index of ’the insurance type used by the respondents’ is the smallest, which are 1105 and 23, respectively.
  The influence indexes of ’Salary level’, ‘ability to judge relevant health information in the media’, ‘probability of medical attendance’, ‘knowledge about vaccinations and checkups’, and ’ability to obtain the healthy eating information’ are the second, the third, the forth, the fifth, and the sixth, respectively.
  The influence indexes of ’Gender’, ’Territory’, ’Education background’, and ‘Professional’ on the HL levels are 69, 96, 69, and 71, respectively.
  The fourth dimension (health status and disease history) of the HL questionnaire is reflected by the $Column_{-}3,4,5,7,8,9,10$ in Fig. 6, where the impact index of ’ health status’ is the largest, which is 168. These results reveal that the forth dimension (health status and disease history) shouldn’t be ignored during investigating HL assessment problems, which provides a new idea for the existing HL questionnaire design with three dimensions.

According to the above analysis, it also can be seen that all questions in the designed questionnaire are reasonable, because that they affect the HL assessment results by varying degrees.

## Discussion

HL is an essential factor that affects health [26]. People with low HL have low self-management skills [27]. Poor HL can also lead to high health care costs. This paper aims at that an exclusive HL assessment questionnaire and LGBM model for Mongolians in China are presented to improve the Mongolians’ HL level assessment accuracy and to find influence factors on HL by analyzing quantitatively every questions, which can provide a new idea for the HL assessment of other ethnic minorities in China or ethnic minorities in other countries.

Four dimensions are considered during the HL questionnaire’s design, which are health concepts and knowledge literacy, healthy lifestyle and behavior, and healthy skills, as well as health status and disease history. It is different from the existing three dimensions methods [28–30] and five dimensions approach [31] in China, because the health status and disease history of respondents aren’t considered in [28–31]. The HL questionnaire with 68 questions are designed by both improving the HLS-EU-Q47 and analyzing the characteristics in Mongolians in China. In order to verify the presented HL assessment method by a set of cross - sectional data, 742 Mongolians in Inner Mongolia of China are invited to answer the above HL questionnaire.

Based on the HL questionnaires completed by 742 Mongolians, the reliability and validity of the designed HL questionnaire are analyzed by using Cronbach’s $\alpha$ coefficient, Mutual Information Score (MIS), KMO and Bartlett Spherical Test Chi-square Value (BSTCV). The results show that the designed HL questionnaire has the high reliability and validity, because we get Cronbach’s $\alpha =0.807$, MIS=0.803, KMO=0.765, and BSTCV=2486 ($p<0.001$) by using our Python programs. The MIS method is better than Pearson correlation coefficient approach [32], because the latter can only handel linear correlations, however, the former can not only deal with linear correlation but also nonlinear correlation.

A data set with 742 samples is constructed, where each sample has 68 features and 1 target. 68 features correspond to 68 questions in the HL questionnaire, and 1 target corresponds to the HL score that each respondent obtained by answering the questionnaire. Based on this data set, the XGB and LGBM regression models to predict HL are constructed, respectively. 80% samples in the above data set are designed as training samples, and others are looked as testing samples. The XGB and LGBM regression models are trained by 594 (80%) samples, respectively. Then the XGB and LGBM regression models are tested by 148 (20%) samples, respectively. The $R^2 (0 <R^2\le 1 )$ index is chosen as an evaluation accuracy index. The large $R^2 (0 <R^2\le 1 )$ means the high assessment accuracy. The results show that $R^2$ index and the absolute error by using LGBM regression model are 0.98347 and 11, respectively, which are better than ones by applying XGB. It can be seen that the HL assessment model based on LGBM can achieve the assessment results with high accuracy.

In addition, the existing correlation analysis methods, such as Covariance method, Pearson correlation coefficient, and MIS approach, can only give quantitative results for analyzing the correlation problem among questions of questionnaires. This does not meet the growing demand for HL assessments with high-precision. Therefore, we quantitatively analyze the influence of each question in the questionnaire on the HL assessment results by using the feature-importance function in the HL assessment model based on LGBM. The quantitative results for correlation analysis among all questions are given in Fig. 6. It can be seen that the biggest impact factor is 1105, and the smallest impact factor is 23. The age has the highest influence on the HL level. It shows there is a strong correlation between age and HL levels, which is consistent with other studies [28–31, 33]. For example, Japanese HL survey [33] concluded that the HL level for Japanese increased with age; The HL survey in European countries and Turkey demonstrated that older people tended to have lower HL [33]. The impact index of the salary level of the respondents ($Column_{-}27$) is 286, which is the second, but it is much smaller than one of age. This result is consistent with the conclusions from [28–30]. The impact index of the ability of the interviewees to judge relevant health information in the media ($Column_{-}36$) is 270, which is the third. The impact indexes of the probability of medical attendance ($Column_{-}25$), the knowing about vaccinations and checkups ()$Column_{-}43$, and the obtaining healthy eating information($Column_{-}53$ ) are the forth, the fifth, and the sixth, which are 256,254, and 253, respectively. These analysis aren’t found in the existing results. The influence of Gender ($Column_{-}1$) on the HL level is 69. The scores of the respondents show that Men’s HL is higher than Women’s HL, which is consistent with ones in [29, 34], but the quantification of influencing factors wasn’t investigated in [29, 34]. The impact indexes of the Territory ($Column_{-}2$), Education background ($Column_{-}20$), and Professional ($Column_{-}21$) are 96, 69, and 71, respectively. And the scores of the respondents show that the HL levels of respondents living in cities are higher than ones of the residents in villages; there is a positive linear correlation between the level of HL and the educational background of the respondents. These results for Territory and Education background are consistent with ones in [29]. The fourth dimension (health status and disease history) of the HL questionnaire is reflected by the $Column_{-}3,4,5,7,8,9,10$ in Fig. 6, where the impact index of the health status ($Column_{-}3$) is the largest, which is 168. However, they aren’t considered in [28–30]. The impact indexes of other questions aren’t addressed individually, which can be found in Fig. 6. It is worth mentioning that the least influence question on the final HL assessment result is the insurance type ($Column_{-}6$), and its value is 23. However, this factor isn’t investigated in other papers.

From Fig. 6 and the above discussion, it can be seen that the designed questionnaire is reasonable, because there are no the features that do not contribute to the health literacy assessment. It is worth mentioning that the HL assessment LGBM model and the quantitative analysis method for each question are suitable for the HL assessment for anyone else.

## Conclusions

The presented HL questionnaire with 68 questions has the high reliability and validity, which are verified by using Cronbach’s $\alpha$, MIS, KMO, and BSTCV theories. The HL level assessment model based on LGBM can assess accurately the HL levels of Mongolians in China. The impact of each question in the questionnaire on the final assessment results can be quantified by using the ‘feature-importance’ function in LGBM model, which is better than the existing qualitative analysis methods. It is worth mentioning that The HL level assessment model based on LGBM and the quantitative calculation based on ‘feature-importance’ method for the influence index of each question on the final assessment results can also be applied to other assessment studies based on scales or data sets.

## Data Availability

The datasets generated and/or analyzed during the current study are not publicly available due to the respondents’ privacy concerns, but are available from the corresponding author on reasonable request.
